# Transcriptomic gene signatures measure satellite cell activity in muscular dystrophies

**DOI:** 10.1016/j.isci.2024.109947

**Published:** 2024-05-08

**Authors:** Elise N. Engquist, Anna Greco, Leo A.B. Joosten, Baziel G.M. van Engelen, Christopher R.S. Banerji, Peter S. Zammit

**Affiliations:** 1 King’s College London, Randall Centre for Cell and Molecular Biophysics, New Hunt’s House, Guy’s Campus, London SE1 1UL, UK; 2 Department of Neurology, Donders Institute for Brain, Cognition and Behaviour, Radboud University Medical Center, Nijmegen 6525 GA, the Netherlands; 3 Department of Internal Medicine, Radboud Institute of Molecular Life Sciences (RIMLS) and Radboud Center of Infectious Diseases (RCI), Radboud University Medical Center, Geert Grooteplein Zuid 10, Nijmegen 6525 GA, The Netherlands; 4 Department of Medical Genetics, Iuliu Hatieganu University if Medicine and Pharmacy, 400012 Cluj-Napoca, Romania; 5 The Alan Turing Institute, The British Library, 96 Euston Road, London NW1 2DB, UK; 6 University College London Hospitals, NHS Foundation Trust, London NW1 2BU, UK

**Keywords:** musculoskeletal medicine, disease, specialized functions of cells, transcriptomics, model organism

## Abstract

The routine need for myonuclear turnover in skeletal muscle, together with more sporadic demands for hypertrophy and repair, are performed by resident muscle stem cells called satellite cells. Muscular dystrophies are characterized by muscle wasting, stimulating chronic repair/regeneration by satellite cells. Here, we derived and validated transcriptomic signatures for satellite cells, myoblasts/myocytes, and myonuclei using publicly available murine single cell RNA-Sequencing data. Our signatures distinguished disease from control in transcriptomic data from several muscular dystrophies including facioscapulohumeral muscular dystrophy (FSHD), Duchenne muscular dystrophy, and myotonic dystrophy type I. For FSHD, the expression of our gene signatures correlated with direct counts of satellite cells on muscle sections, as well as with increasing clinical and pathological severity. Thus, our gene signatures enable the investigation of myogenesis in bulk transcriptomic data from muscle biopsies. They also facilitate study of muscle regeneration in transcriptomic data from human muscle across health and disease.

## Highlights

• Novel gene signatures for assessing myogenesis in bulk muscle transcriptomic data
• Satellite cell activity increased in muscle biopsies from facioscapulohumeral MD
• Satellite cell activity increased in Duchenne MD and Myotonic dystrophy type 1

## Graphical abstract

**Figure:**
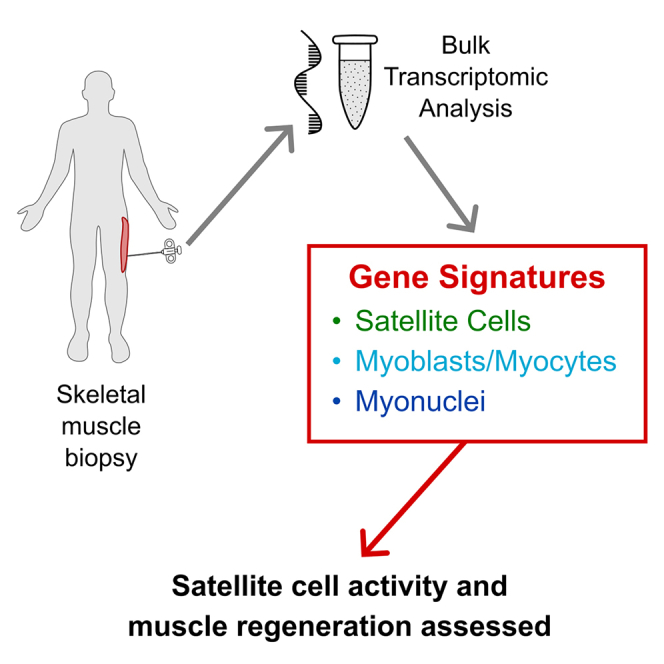

## Introduction

Skeletal muscle is an archetypal adult stem cell model. The functional unit of skeletal muscle is the myofiber, a large syncytial cell with hundreds/thousands of post-mitotic myonuclei. Routine needs for myonuclear homeostasis, and the more sporadic demands for hypertrophy and repair, are effectuated by resident muscle stem cells termed satellite cells.^1^^,^^2^ Satellite cells reside between the sarcolemma and basal lamina of myofibres^3^ and are typically mitotically quiescent in mature muscle.^4^ Upon stimulation, for example following strenuous exercise, injury or disease, satellite cells are activated and undergo extensive proliferation to generate myoblast progeny, which eventually either differentiate to provide new myonuclei or self-renew to maintain the stem cell pool.^1^^,^^5^^,^^6^^,^^7^ While additional populations of muscle-resident cells such as fibro-adipogenic precursors (FAPs)^8^ and macrophages^9^ assist in the regulation and coordination of muscle regeneration, satellite cells are the source of new myonuclei.^1^

Muscular dystrophies are genetic diseases characterized by muscle wasting, meaning that the repair/regeneration conducted by satellite cells becomes progressively compromised.^10^ Mutations in genes key to satellite cell function such as *PAX7*, *MYOD1,* and *MYMK* have been associated with specific forms of muscle disease.^11^^,^^12^^,^^13^^,^^14^ With respect to the involvement of satellite cells in disease progression, we have recently introduced the concept that muscular dystrophies can be classified as 1) “Primary satellite cell-opathies” in which the causative mutation exclusively affects satellite cell function, 2) “Secondary satellite cell-opathies” in which the pathogenic mutation affects both satellite cells and muscle fibers or 3) “Non-satellite cell-opathy neuromuscular disorders” in which satellite cell function is not directly affected by the causative mutation, however satellite cells are operating in an increasing hostile microenvironment that indirectly affects their ability to maintain muscle.^15^^,^^16^ Diseases in which satellite cells are compromised by environmental factors can be added to this classification.^17^

Considering the contribution of satellite cell dysfunction to muscle disease, it is important to have means to measure satellite cell activity in human muscle. Gene expression changes in satellite cells, myoblasts, and myocytes throughout myogenesis have been widely explored^18^^,^^19^^,^^20^^,^^21^^,^^22^^,^^23^ using *in vitro*/*ex vivo* cell and myofiber cultures systems, as well as animal models to study injury and transplantation. However, while animal models offer diverse methodology to investigate muscle growth and repair, such methods are not feasible in humans, meaning the investigation of satellite cells in human muscle *in situ* therefore generally relies on samples of bulk muscle tissue biopsies. Human muscle biopsies can be used in a number of ways including 1) the isolation of primary satellite cells/myogenic precursors/muscle fibers to examine their functional capabilities *ex vivo*, 2) histological analysis of tissue sections (i.e., for the presence of activated/proliferating satellite cells, regenerating fibers expressing developmental myosin heavy chain isoforms, and regenerated fibers containing centrally located myonuclei) and/or 3) transcriptomic/proteomic analyses of tissue samples. These methods have provided valuable insight into regeneration, however are often limited by factors such as time and labor intensiveness and tissue availability, and as such bulk RNA-sequencing has become an appealing option in recent years due to its increased affordability and the large number of genes analyzed from a relatively small input sample. However, this too has caveats when considering satellite cell and myogenic precursors, as gene expression changes identified in muscle biopsies are a composite signal from multiple cell types, of which satellite cells are only a small fraction. As such, sets of genes whose change in expression indicate ongoing regeneration are often of limited use as cell specific markers in the bulk tissue setting. Increased accessibility of single-cell and single-nucleus technologies now allow more in-depth investigations of individual cell populations.^24^ Recent single-cell RNA-sequencing of individual human muscles^25^^,^^26^ have provided sufficient resolution to investigate satellite cells in human muscle, however cells actively undergoing myogenesis are infrequently captured due to their sparsity.

In this study, we established three novel gene sets, one specific for satellite cells, another for myoblasts/myocytes, and the last for myonuclei, whose expression is largely restricted and so can be used to inform on these cell populations in transcriptomic data from a bulk tissue sample. The sets of genes were derived using the amalgamated murine single cell/nucleus RNA-sequencing data compiled and analyzed by McKellar et al.^24^ They were then optimized and validated in two independent human muscle single cell transcriptomic datasets, as well as by directly comparing their expression in human bulk RNA-sequencing data to histological analysis of paired tissue sections from the same muscles. Our novel Satellite Cell Signature, Myoblast/Myocyte Signature and Myonuclear Signature distinguished disease from control in transcriptomic data describing several muscular dystrophies including facioscapulohumeral muscular dystrophy (FSHD), Duchenne muscular dystrophy (DMD) and myotonic dystrophy type I (DM1), consistent with muscle regeneration reported in those conditions.^27^ For FSHD, multimodal datasets^28^^,^^29^ revealed the correlation of gene signature expression with worsening clinical and pathological severity. These gene signatures provide tools to investigate the activity of various myogenic populations in bulk RNA-sequencing data from human muscle biopsies. They will facilitate further investigation of muscle regeneration in transcriptomic data from human muscle across health, disease, age, exercise, and following therapeutic intervention.

## Results

### Generation of gene signatures for satellite cells, myoblasts/myocytes, and myonuclei

**Figure 1:**
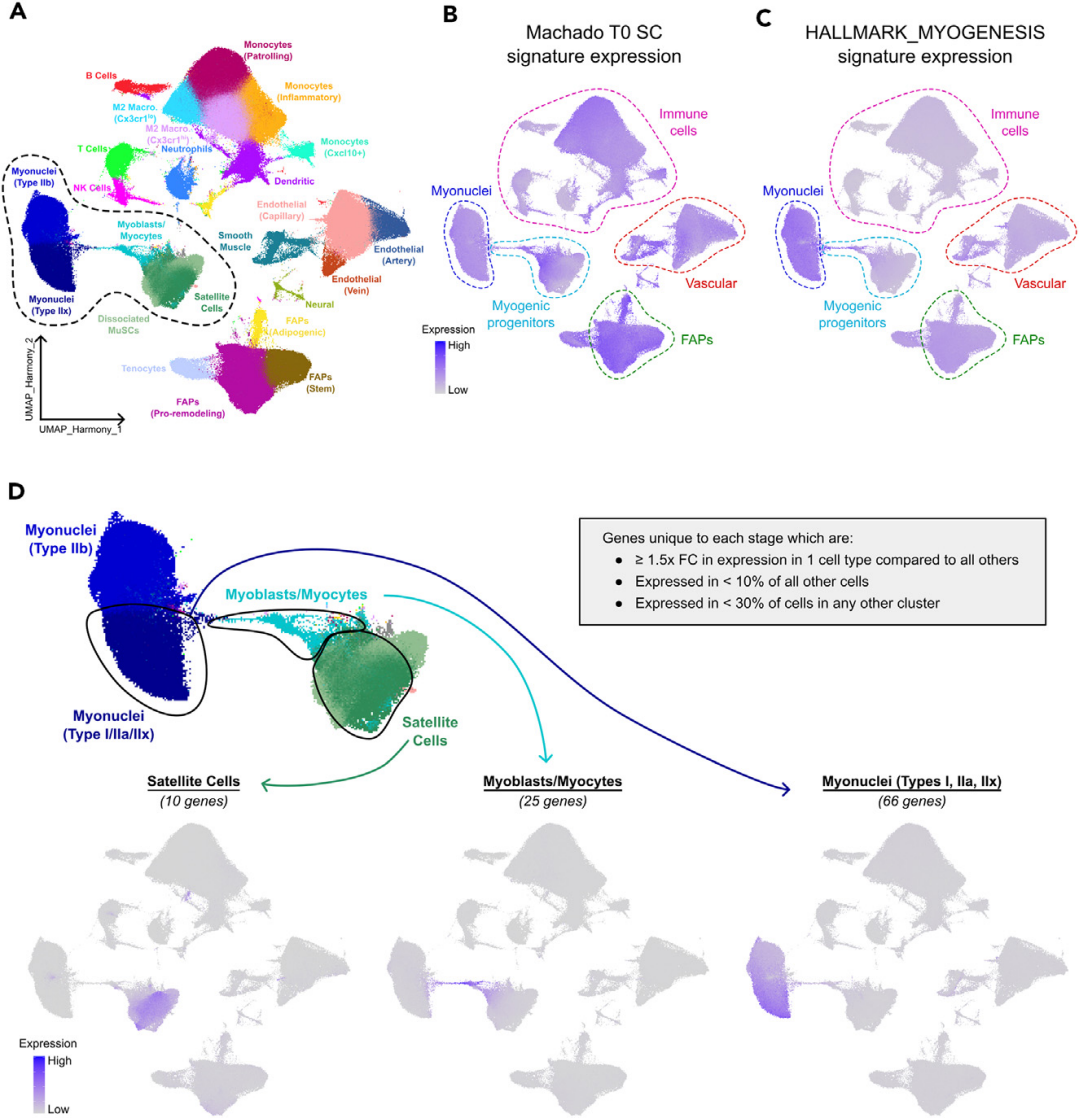
Generation of gene signatures unique to specific stages of myogenesis (A) UMAP projection of mouse single-cell data adapted from McKellar et al.,^24^ consisting of 365,011 cells. Myogenic precursor populations used when generating gene signatures are highlighted with a dashed line. (B) UMAP plot of McKellar et al.^24^ dataset from (A) in which coloring of cells represents the expression of the 4113 genes Machado et al.^19^ identified as up-regulated in murine quiescent satellite cells relative to satellite cells in early stages of activation with FC > 2 and *p* < 0.05. Expression represents sum of the log-transformed counts of all 4113 genes within a cell. Clusters from (A), grouped broadly by cell identity, are enclosed by colored dashed lines. (C) UMAP plot of McKellar et al.^24^ dataset from (A) in which coloring of cells represents the expression of the 199 genes comprising the HALLMARK_MYOGENESIS^30^ from the Molecular Signatures Database (MSigDB). Expression represents the sum of the log-transformed counts of all 199 genes within a cell. Clusters from (A), grouped broadly by cell identity, are enclosed by colored dashed lines. (D) Identification of genes uniquely expressed in different stages of murine myogenesis. Top UMAP plot shows myogenic cells from (A), with dashed lines highlighting sub-populations of myogenic precursor populations used to generate gene signatures, including satellite cells (dark green, 27586 cells), myoblasts, and myocytes undergoing myogenesis (turquoise, 3685 cells), and type I/IIa/IIx myonuclei (dark blue, 18601 nuclei). Signatures consist of differentially expressed genes that exhibit a >1.5-fold increase in expression in the given sub-population and are expressed in <10% of all other cells and <30% of all cells within any other individual cluster.^24^ Bottom three UMAP plots depict the expression of the Satellite Cell (10 genes), Myoblast/Myocyte (25 genes) and Myonuclear (66 genes) Signatures across all cell types in the McKellar dataset. Cells are colored by signature expression, calculated as the sum of the log-transformed counts of genes in a signature within each cell.

Transcriptomic changes throughout myogenesis have been widely explored *in vitro/ex vivo*^18^^,^^19^^,^^20^^,^^21^^,^^22^^,^^23^ but their suitability to investigate satellite cell activity in bulk tissue transcriptomic data is debatable given that satellite cells and myogenic precursors usually represent only a small fraction of total cells. A valuable new resource is the large composite murine skeletal muscle single-cell/single-nucleus transcriptomic dataset available from McKellar et al. 2021,^24^ consisting of 365,011 muscle tissue cells of which 84,383 are myogenic (Figure 1A). We evaluated the specificity for satellite cells of two published gene lists: the first comprising genes that simply significantly change expression during murine satellite cell activation,^19^ and the second being the Molecular Signatures Database “HALLMARK_MYOGENESIS” gene signature.^30^ Both gene sets were clearly expressed in multiple myogenic populations in mouse, however they were also expressed in other cell types present in bulk muscle tissue (Figures 1B and 1C).

To generate sets of genes that each characterize different myogenic cell populations, we used the valuable resource provided by McKellar et al.,^24^ as this integrated murine dataset consists of multiple cell types including satellite cells, myoblast/myocytes and the three main subtypes of myonuclei expressed in human muscle (type I, IIa and IIx) (Figure 1D). We optimized a series of selection parameters (see Methods) to identify 10 genes with expression restricted to satellite cells, hereby termed the “Satellite Cell Signature,” 25 genes in myoblasts/myocytes termed the “Myoblast/Myocyte Signature” and 66 genes largely limited to type I/IIa/IIx myonuclei called the “Myonuclear Signature” (Figure 1D). The genes comprising each signature are listed in Table 1, with their roles/functions and mouse homologues detailed in Table S1.

**Table 1:** Genes in the satellite cell, myoblast/myocyte, and myonuclear signatures

Satellite cell signature (10 genes)	Myoblast/Myocyte signature (25 genes)		Myonuclear (Type I/IIA/IIX) signature (66 genes)				
*CHRDL2*	*BEX1*	*MYMX*	*ABCB4*	*CLASP2*	*ITGB6*	*PDK4*	*SORBS1*
*CLCF1*	*CAPN6*	*MYOG*	*ACSS2*	*COQ8A*	*IVD*	*PFKFB1*	*SYNJ2*
*CRLF1*	*CAV3*	*PNMA8B*	*ADAMTSL5*	*CRHR2*	*KCNA7*	*PPARGC1A*	*SYNPO2L*
*DHCR24*	*CHRNA1*	*POPDC3*	*AGBL1*	*CSRP3*	*KCNJ2*	*PPARGC1B*	*TBX1*
*ERFE*	*FNDC5*	*RAPSN*	*AKAP1*	*DMAC2*	*KCNN2*	*PPFIBP2*	*TMEM52*
*FOSL1*	*GPC1*	*RTN2*	*AKAP6*	*DNAJB5*	*KCNQ4*	*PRICKLE3*	*USP28*
*MYF5*	*IFFO1*	*SCRIB*	*ANKRD2*	*DUSP18*	*LMOD2*	*PTPN3*	*XIRP1*
*NPPC*	*IQSEC3*	*SYTL2*	*ANKRD52*	*FABP3*	*LMOD3*	*RILP*	*YIPF7*
*PAX7*	*LRRN1*	*TNNC1*	*ANO5*	*FEM1A*	*LRPPRC*	*RMND1*	*ZCCHC4*
*PDE10A*	*MEGF10*	*TNNI1*	*AOX1*	*FSD2*	*MAPK1IP1L*	*RPS6KA2*	*ZNF17*
	*MYH3*	*TNNT1*	*APOL6*	*GMPR*	*MYH2*	*SACS*	
	*MYL4*	*TNNT2*	*ASB10*	*GPR157*	*MYOZ2*	*SLC25A12*	
	*MYMK*		*ATP1B4*	*HOMER2*	*NOS1*	*SLF1*	
			*C10orf71*	*HSPB7*	*PADI2*	*SMTNL1*	

### Expression of satellite cell, myoblasts/myocyte, and myonuclear signatures correlate with appropriate stages of rodent muscle regeneration

**Figure 2:**
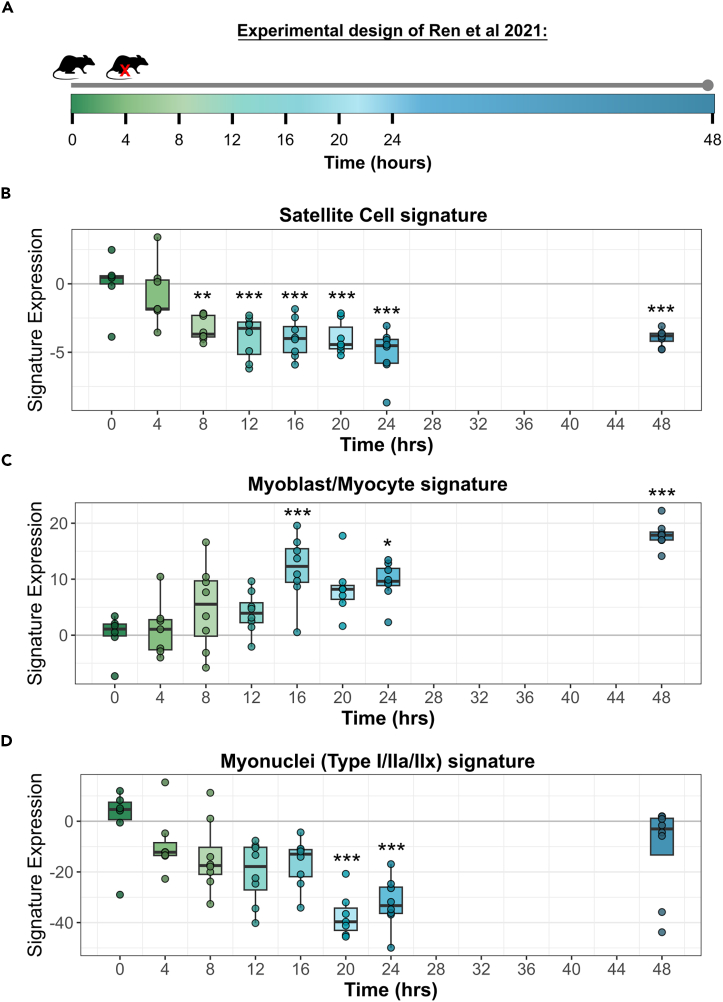
The Satellite Cell, Myoblast/Myocyte, and Myonuclei Signatures correlate with early events in rodent muscle regeneration (A) Experimental overview of Ren et al.^31^ RNA-sequencing data. Rats were randomly divided into experimental groups and hindlimb muscle samples were collected for transcriptomic analysis from either non-injured muscle (0 h) or muscle after 4, 8, 12, 16, 20, 24, or 48 h post injury. (B–D) Boxplots show the expression of the Satellite Cell, Myoblast/Myocyte, and Myonuclei Signatures in the Ren et al.^31^ rodent dataset. Raw counts were obtained from Gene Expression Omnibus (GSE171243) and normalized in DESeq2. Expression scores for each sample were calculated as the sum of the log-scaled counts of all genes in a signature after the subtraction of the time 0 h control average. Boxes represent the interquartile range (IQR), lines display the median, and whiskers depict the smallest and largest values within 1.5∗IQR from the IQR. Significance was determined by one-way ANOVA with Tukey’s post-hoc test. Asterisks denote significance relative to the 0 h control group, with one asterisk representing *p* < 0.05, two asterisks *p* < 0.01, and three asterisks *p* < 0.001.

To assess the ability of our three gene signatures to detect changes in myogenic sub-types in bulk transcriptomic data, we first examined their expression during rodent muscle regeneration (Figure 2). We used the time series transcriptomic dataset from Ren et al.^31^ (GSE171243) in which muscle samples were collected from the hindlimb of non-injured rats, and then at 4 h intervals for 24 h after weight-drop injury (Figure 2A). Expression of the Satellite Cell Signature was significantly reduced as early as 8 h post-injury and remained suppressed at the final 48-h timepoint (Figure 2B), while the expression of the Myoblast/Myocyte Signature significantly increased from 16 h following injury and then remained elevated through to 48 h (Figure 2C). Together these findings are consistent with known fluctuations in satellite cell and myoblast/myocyte proportions following injury. Expression of the Myonuclear Signature declined significantly by 20 h post-injury but recovered by 48 h (Figure 2D), consistent with qualitative observations of partial muscle repair by 48 h reported by the authors.^31^

### Satellite cell and myonuclei signatures identify respective cell populations in human muscle

**Figure 3:**
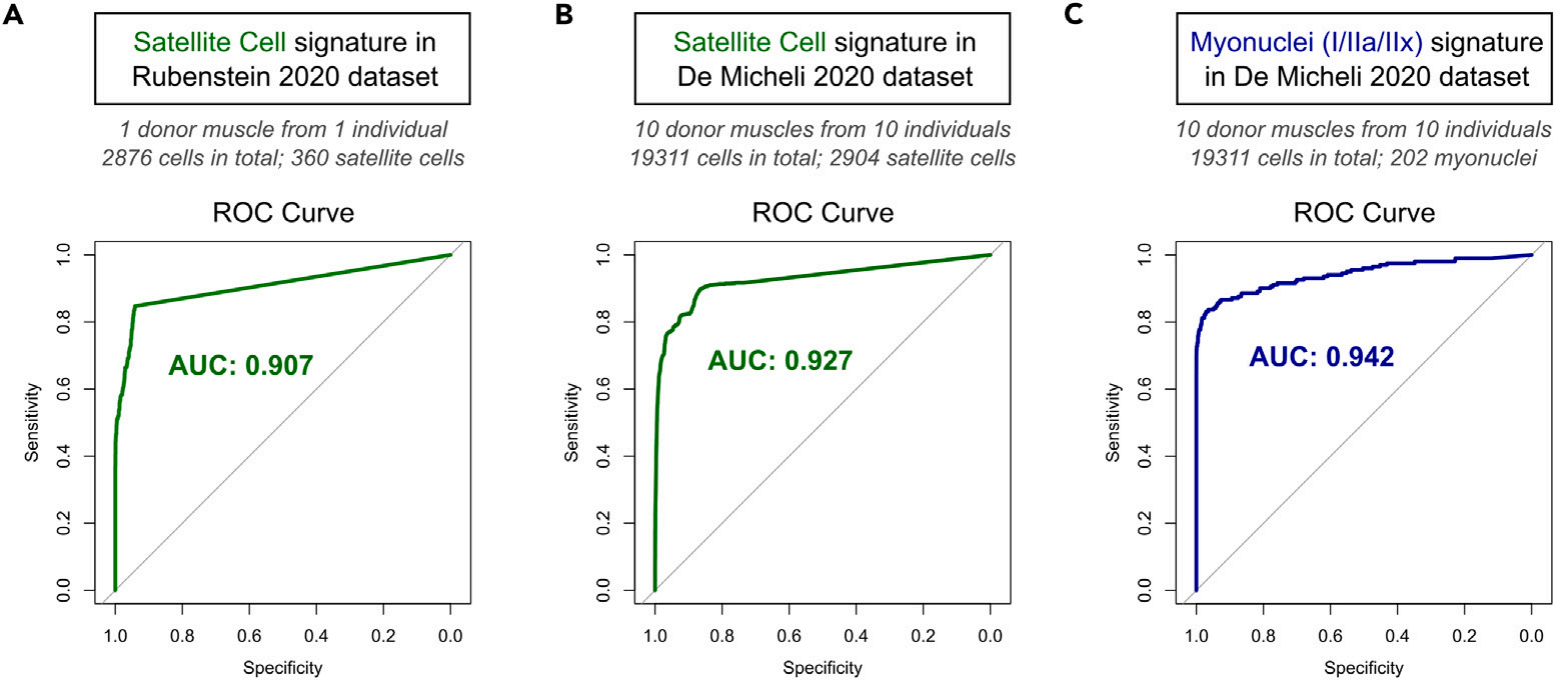
The Satellite Cell and Myonuclear Signatures identify satellite cells and myonuclei in human muscle Expression of Satellite Cell signature (10 genes) and Myonuclear signature (66 genes) in human single cell transcriptomic data from (A) Rubenstein et al. 2021^25^ and (B and C) De Micheli et al. 2020.^26^ (A and B) ROC curves display the discriminatory power of the Satellite Cell Signature in each dataset, with high AUC values indicating that the signature is a very good classifier of satellite cells in human muscle. (C) ROC curve displays the discriminatory power of the Myonuclear signature in the De Micheli dataset,^26^ with the high AUC value showing that this signature is a powerful classifier of myonuclei in human muscle.

As these gene signatures were developed using murine data, we also sought to examine their expression in human muscle (Figure 3). Two independent human scRNA-sequencing datasets were examined: Rubenstein et al.^25^ (GSE130646) consisting of 2876 cells from the vastus lateralis of a young healthy male donor and De Micheli et al.^26^ (GSE143704) with ∼19,000 cells from 10 different muscles from 10 individuals of mixed genders and ages. Both datasets contained satellite cells, and De Micheli et al.^26^ also contained a small number of residual myonuclei. We used receiver operating characteristic (ROC) curve analysis to evaluate the expression of the Satellite Cell Signature as a binary classifier of satellite cell status in each human dataset. The area under the curve (AUC), representing the average discriminatory power of the signature, was greater than 0.90 in both datasets (Figures 3A and 3B), establishing the Satellite Cell Signature as an accurate classifier of satellite cells in single cell data from human skeletal muscle. ROC curve analysis of the Myonuclear Signature in the De Micheli et al.^26^ dataset also performed very well, with an AUC of 0.942 (Figure 3C). Similar analyses were not possible for the Myoblast/Myocyte Signature, as neither dataset contained enough myoblasts/myocytes to form their own cluster, as expected for healthy mature human muscle.

### Satellite cell signature expression correlates with the histological quantification of PAX7-containing cells

**Figure 4:**
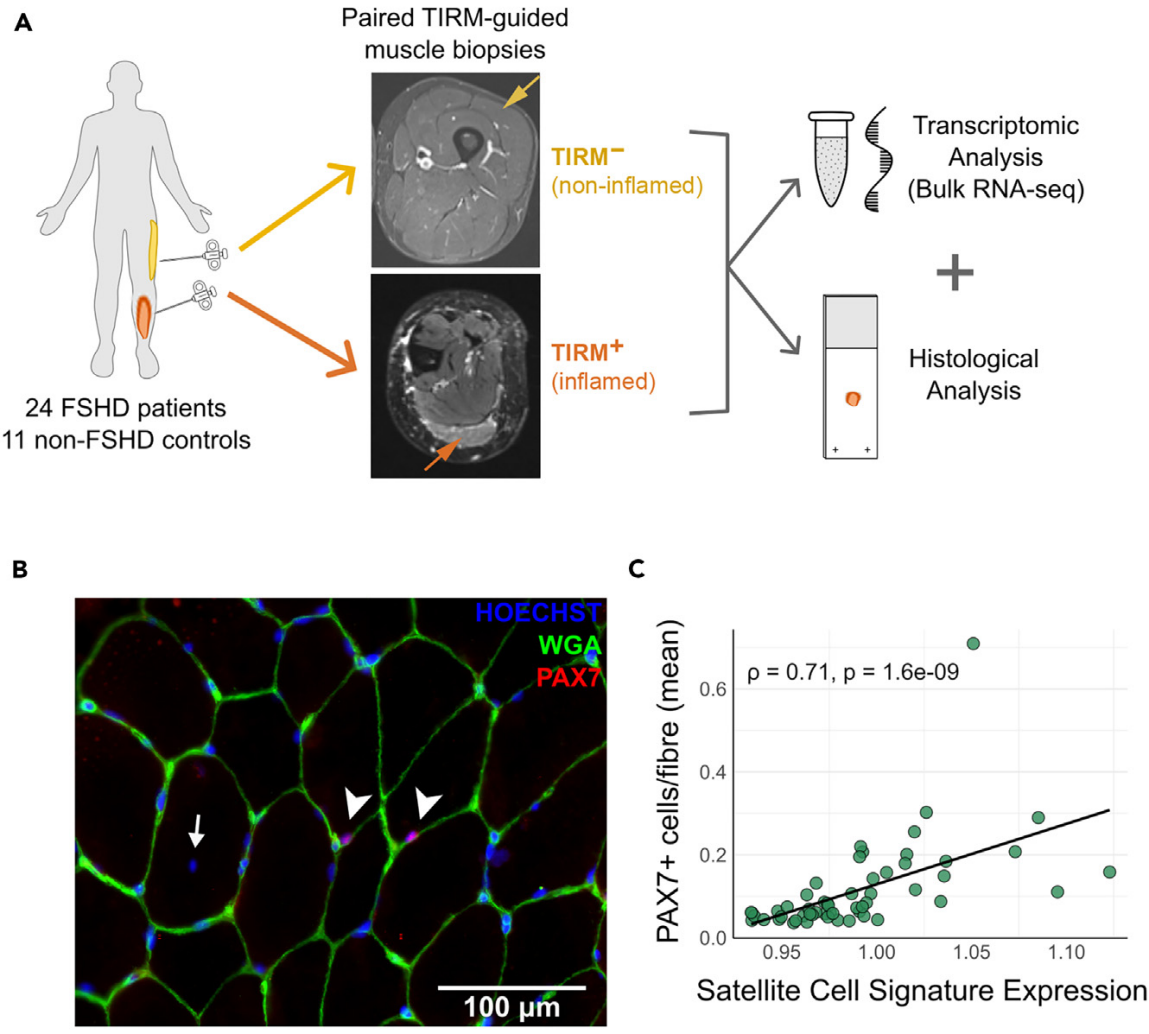
Satellite Cell, Myoblast/Myocyte, and Myonuclear Signature expression correlates with the histological quantification of satellite cells (A) Overview of paired bulk RNA-sequencing and histology muscle biopsy samples from patients with FSHD reported in Banerji et al.^28^ Twenty-four patients with FSHD donated biopsies from both a non-inflamed (TIRM^–^) muscle and an inflamed (TIRM^+^) muscle, as determined by MRI-guidance. Biopsies were also collected from eleven healthy control individuals. Each biopsy was then divided for bulk RNA-sequencing and sectioning for histological analysis, with the exception of 3 controls in which only RNA-sequencing was performed, thus excluding them from this analysis. Two TIRM^+^ samples were excluded from the analysis due to an insufficient number of myofibres. (B) Representative image of PAX7 immunolabelling (red) of FSHD patient muscle to identify satellite cells, counterstained with Hoechst33342 (blue) to highlight all nuclei and wheat germ agglutinin (green) to delimit myofibres. Scale bar is approximately 100 μm. Sections were imaged with a slide scanner and PAX7-containing cells quantified from the entire sections. (C) Graph depicts the Satellite Cell Signature expression plotted against the mean number of PAX7+ satellite cells per myofiber in each sample. Signature expression score represents the sum of log-transformed counts of genes within the signature scaled across samples. Each point represents data from a single biopsy. Regression lines are presented are Spearman correlation co-efficient (ρ) and *p*-value in black text.

We also examined the relationship between the expression of our Satellite Cell Signature and the number of PAX7-containing cells determined histologically, using paired RNA-sequencing data and tissue sections collected from the same human muscle biopsy samples. This was undertaken using our recently published dataset in which muscle biopsies were collected from 11 healthy individuals and 24 individuals with FSHD,^28^ a condition in which muscle regeneration has been reported.^27^ As described in Banerji et al. 2023^28^ and Engquist et al. 2024,^32^ each FSHD patient donated biopsies from two muscles selected on turbo inversion recovery magnitude (TIRM) imaging: one from the non-inflamed vastus lateralis (23/24) muscle (TIRM^–^) and one from an inflamed (TIRM^+^) muscle, and the healthy individuals donated a single biopsy from the vastus lateralis (Figure 4A). These biopsies were then used for bulk RNA-Sequencing and cutting muscle sections (Figure 4A).

Muscle tissue sections from both FSHD and controls were immunolabelled for PAX7 to identify satellite cells, stained with Hoechst to reveal nuclei and wheat germ agglutinin (WGA) to delimit muscle fibers (Figure 4B). Expression of the Satellite Cell Signature in RNA-sequencing samples showed a positive correlation with the average number of satellite cells (PAX7+ nuclei) per muscle fiber (Figure 4C) in the corresponding muscle sections. To evaluate whether this correlation was due to chance, the expression of 1000 sets of 10 genes selected through random resampling were correlated with the number of PAX7+ cells observed histologically, and none out-performed the Satellite Cell Signature (*p* < 0.001).

### Expression of the satellite cell and myoblast/myocyte signatures are elevated in facioscapulohumeral muscular dystrophy

Finally, we examined the expression of these new gene signatures across three types of human muscular dystrophy: FSHD, DMD, and DM1.

**Figure 5:**
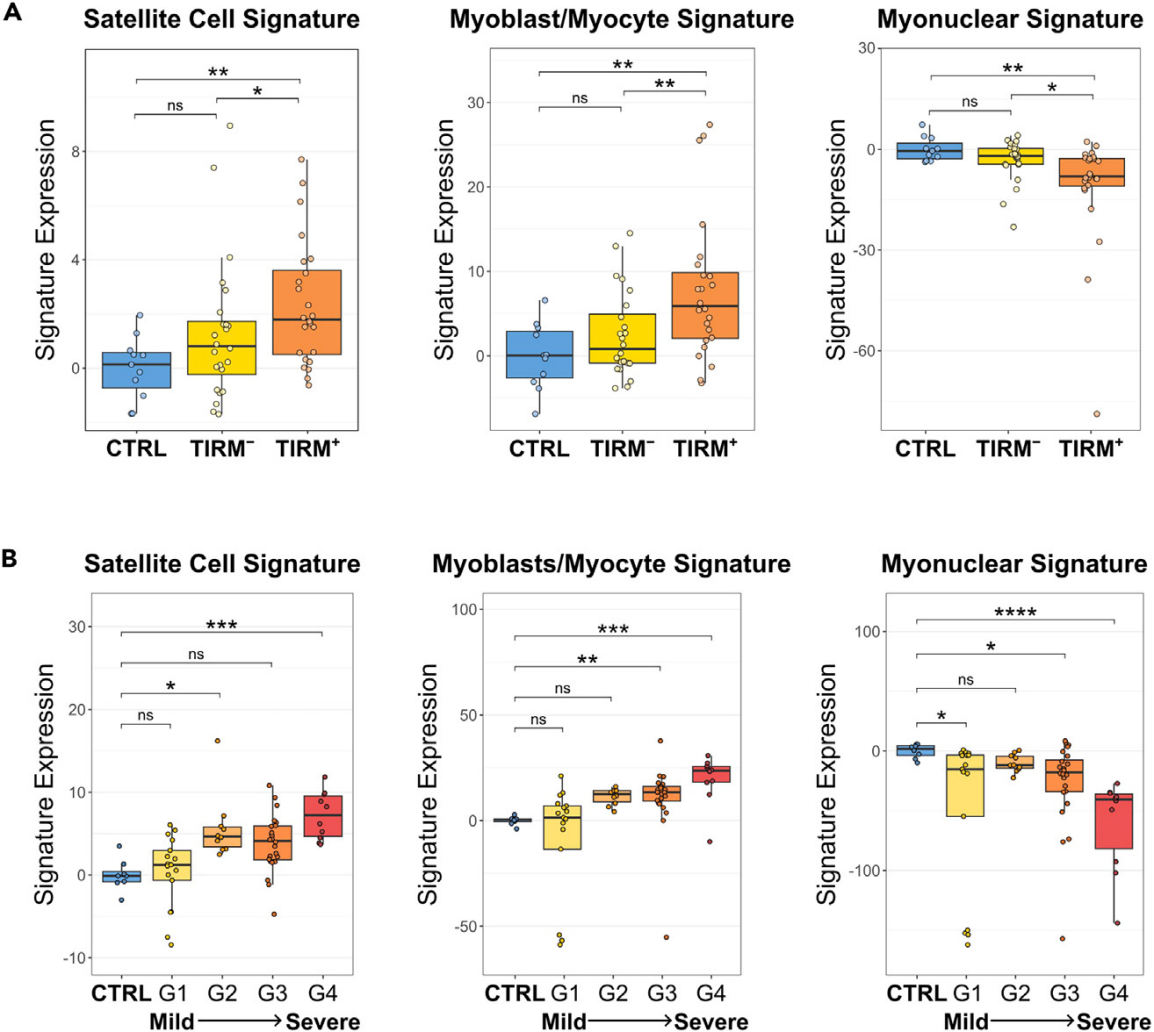
Satellite Cell and Myoblast/Myocyte Signature expression is elevated in FSHD (A) Boxplots show the increased expression of the Satellite Cell and Myoblast/Myocyte signatures but decreased expression of the Myonuclei Signature in inflamed (TIRM^+^, orange) muscle of patients with FSHD compared to TIRM^–^ (yellow) and unaffected individuals (blue) from the Banerji et al. 2023 dataset.^28^ Expression scores were calculated as the difference between the sum of the log-normalized counts of all genes in a signature from the control average. Boxes represent the interquartile range (IQR) with lines showing the median and denoting the smallest/largest values within 1.5∗IQR outside the IQR. Statistical significance was determined by Wilcoxon test (unpaired for TIRM^–^/Ctrl and TIRM^+^/Ctrl, paired for TIRM^–^/TIRM^+^), where a single asterisk denotes *p* < 0.05, two asterisks denote *p* < 0.01, and three asterisks *p* < 0.001. (B) Box-plots show the expression of the three signatures in RNA-sequencing data from an independent FSHD cohort collected from inflamed muscles (as determined by MRI), and graded with respect to the degree of pathology and DUX4 target gene expression, as reported by Wong et al. 2020.^29^ There was an increase in expression of the Satellite Cell and Myoblast/Myocyte signatures with increasing severity. Expression scores were calculated as the difference between the sum of the log-normalized counts of all genes in a signature from the control average. Boxes represent the interquartile range (IQR) with lines showing the median and whiskers denoting the smallest and largest values within 1.5∗IQR. Statistical significance was determined by one-way ANOVA with Tukey’s post-hoc test or Kruskall-Wallis test with Dunn’s post-hoc when normality was not met, where an asterisk denotes *p* < 0.05, two asterisks *p* < 0.01, three asterisks *p* < 0.001, and four asterisks *p* < 0.0001.

Starting with FSHD, we first used our Banerji et al. 2023^28^ human dataset (outlined in Figure 4A) consisting of bulk RNA-Sequencing data from paired non-inflamed (TIRM^–^) and inflamed FSHD (TIRM^+^) muscle biopsies from each of 24 patients with FSHD, together with muscle biopsies from 11 unaffected individuals. Expression of the Satellite Cell and Myoblast/Myocyte Signatures were elevated in inflamed TIRM^+^ FSHD samples compared to both TIRM^–^ FSHD muscle and controls but not when comparing controls and TIRM^–^ FSHD samples (Figure 5A). This is consistent with muscle regeneration demonstrated histologically in muscle biopsies from patients with FSHD.^27^ Myonuclear gene expression was decreased in inflamed FSHD muscle (Figure 5A), presumably due to a combination of muscle wasting and increased proportions of non-myogenic cell populations accompanying inflammation.

A second independent FSHD transcriptomic dataset from Wong et al.^29^ was also examined, consisting of muscle biopsies from inflamed FSHD muscle (as evaluated by MRI guidance) and healthy controls. These FSHD muscle biopsies were separated into four groups (G1-G4) by the authors based on increasing pathological severity as determined by a combination of histological examination and expression of four disease-related (DUX4 target) genes. Expression of the Satellite Cell and Myoblast/Myocyte Signatures increased with pathological severity, while the expression of the Myonuclei gene signature decreased (Figure 5B).

**Figure 6:**
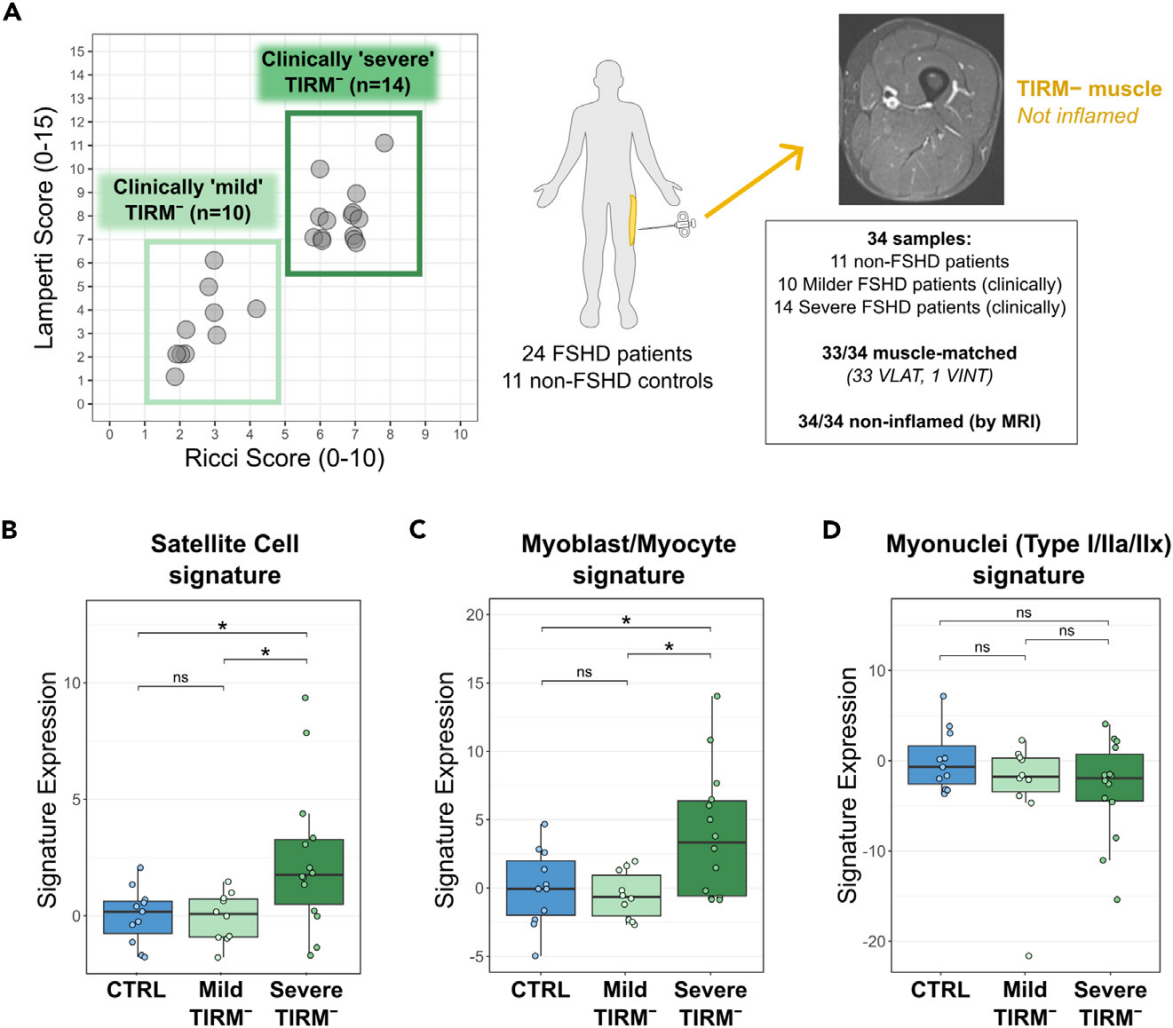
Satellite Cell and Myoblast/Myocyte Signature expression is elevated in non-inflamed muscle of more severely clinically affected patients with FSHD (A) Dot plot shows patient scores for each the two FSHD-specific clinical severity assessments (Ricci Score and Lamperti Score) from the 24 FSHD patient cohort. Each dot represents a patient. Patients segregate into two groups; one group with milder clinical presentation (*n* = 10), and one group more severely affected clinically by disease progression (*n* = 14). Schematic on right summarizes the subset of the dataset stratified by clinical severity that can be used to examine the effects of global disease progression on non-inflamed muscle. (B–D) Boxplots show the expression of the three signatures in control (blue) muscle compared to non-inflamed muscle from patients with either “mild” (light green) or “severe” (dark green) global disease progression from the Banerji et al.^28^ patient cohort. Signature expression represents the sum of the log-normalized counts of genes in a signature within each sample subtracted from the control average. Boxes represent the interquartile range (IQR) with lines showing the median and whiskers denoting the smallest and largest values within 1.5∗IQR. Statistical significance was determined by Wilcoxon ranked-sum test, where an asterisk denotes *p* < 0.05.

The Banerji et al. 2023^28^ dataset also contains the clinical assessment of the patients with FSHD supplying the muscle biopsies. Using the two FSHD-specific clinical severity assessments of the Ricci Score and Lamperti Score, it was possible to segregate the 24 TIRM^–^ FSHD vastus lateralis samples into those from patients that were classified as clinically “mild” (*n* = 10) or “severe” (*n* = 14) (Figure 6A). Assessing the three gene signatures across control, “mild” TIRM^–^ FSHD and “severe” TIRM^–^ FSHD samples demonstrated that expression of the Satellite Cell and Myoblast/Myocyte Signatures also increased with clinical severity, being higher in the “severe” TIRM^–^ FSHD muscle biopsies (Figure 6B - C) compared to the “mild” TIRM^–^ FSHD and controls, while the Myonuclear Signature was unchanged (Figure 6D).

### Satellite cell and myoblast/myocyte signature expression is elevated in Duchenne muscular dystrophy and myotonic dystrophy type I

**Figure 7:**
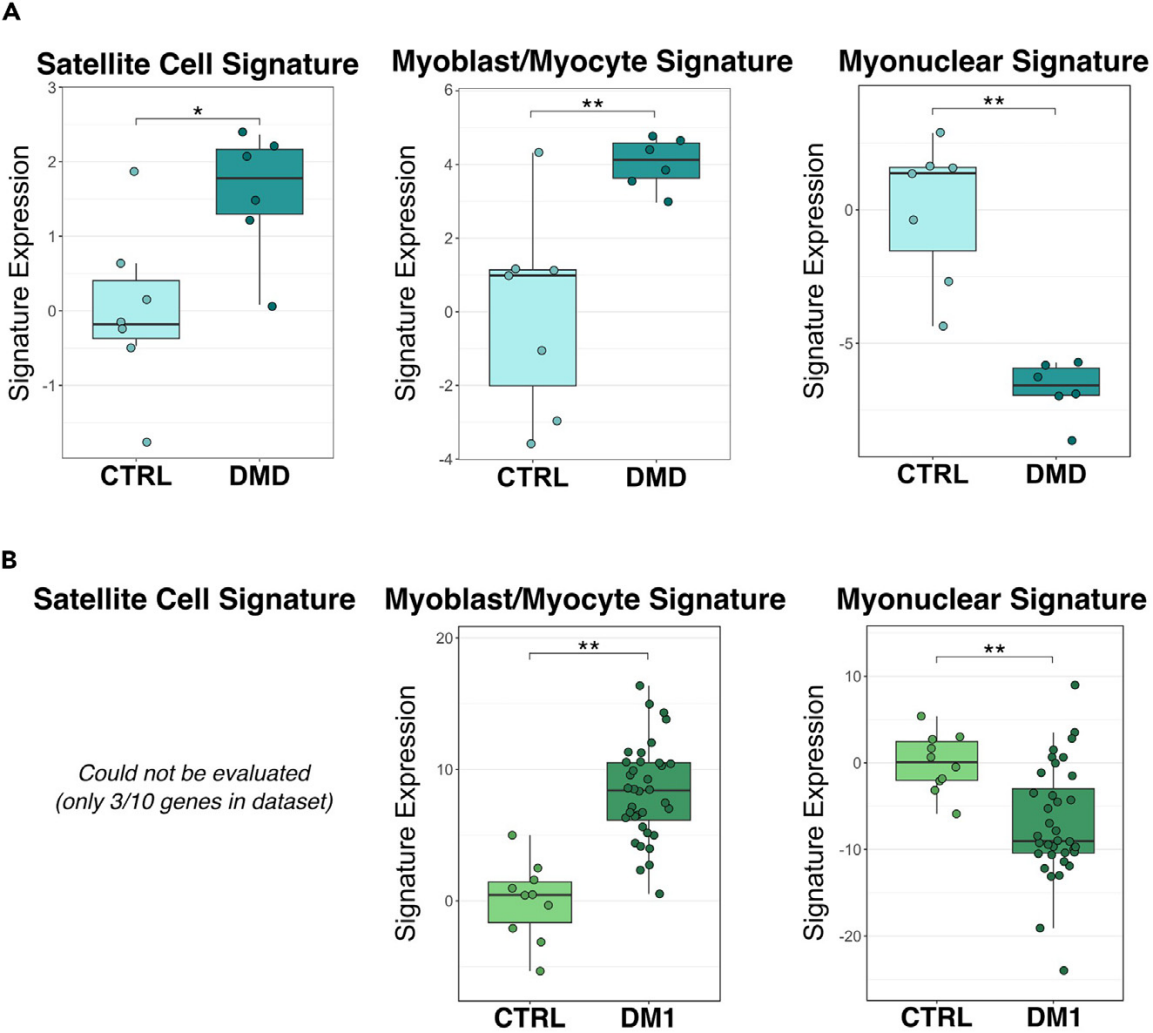
Satellite Cell and Myoblast/Myocyte Signatures indicate increased satellite cell activity in DMD and DM1 muscle (A) Boxplots show the expression of the Satellite Cell, Myoblast/Myocyte, and Myonuclear Signatures in bulk RNA-sequencing data of muscle biopsies collected from 6 control individuals (light blue) and 7 patients with DMD (dark blue), reported by Khairallah et al.^33^ Normalized counts from Table S2 of Khairallah et al.^33^ were used, and expression scores represent the sum of the log-normalized counts of all genes in a signature within a sample subtracted from the control average. Boxes represent the interquartile range (IQR) with lines showing the median. Statistical significance was determined by Wilcoxon ranked-sum test, where an asterisk denotes *p* < 0.05 and two asterisks denote *p* < 0.01. (B) Boxplots show the expression of the Myoblast/Myocyte and Myonuclear Signatures in bulk RNA-sequencing data of muscle biopsies collected from 10 control (light green) or 36 patients with DM1 (dark green), reported by Wang et al.^34^ Using normalized counts from Table S5 of Wang et al.,^34^ and expression scores represent the sum of the log-normalized counts of all genes in a signature within a sample subtracted from the control average. Boxes represent the interquartile range (IQR) with lines showing the median and whiskers denoting the smallest and largest values within 1.5∗IQR. Statistical significance was determined by Wilcoxon ranked-sum test, where two asterisks denote *p* < 0.01.

We also examined publicly available transcriptomic data from human muscle biopsies of patients with DMD and DM1. The DMD dataset consisted of six patients and seven controls (Khairallah et al. Table S2).^33^ The Satellite Cell and Myoblast/Myocyte Signatures were significantly elevated in DMD patient muscle biopsies compared to controls, while the Myonuclear Signature was reduced (Figure 7A).

The DM1 dataset consisted of thirty-six patients and ten controls (Wang et al. Table S5).^34^ While the Satellite Cell Signature could not be analyzed due to insufficient representation of genes in the signature in this dataset, the Myoblast/Myocyte Signature expression was significantly elevated in DM1 patient muscle biopsies compared to controls, and the expression of the Myonuclear Signature was reduced (Figure 7B).

## Discussion

Study of human satellite cells *in situ* can be challenging given their low proportion compared to other cells/nuclei, time-consuming histopathological quantification, and often limited availability of human muscle samples, particularly from patients with ongoing muscle loss. Bulk RNA sequencing generates information on thousands of genes from a relatively small input sample, and has provided valuable in-depth profiling of gene expression throughout myogenesis when performed on purified populations of cells. However, many of the genes identified in these studies are of limited use in a bulk tissue setting due to expression in other cell types. Single-cell and single-nuclei sequencing technologies are informative but remain costly at large scale. Our aim was to generate gene signatures to specific cell types that characterise stages of myogenesis, in order to allow the investigation of satellite cell activity in transcriptomic data from bulk human muscle samples.

To develop new signatures suitable for bulk transcriptomic data, we used the massive repository of murine single cell data compiled from multiple independent studies by McKellar et al.^24^ to identify 10 genes with expression specific to satellite cells, 25 genes specific to myoblasts and myocytes, and 66 genes specific to the types of myonuclei found in human muscle (muscle fiber type I, IIa, and IIx). Each of these signatures includes some expected genes, for example *PAX7* and *MYF5* in the Satellite Cell Signature,^35^^,^^36^ *MYOG*, *MYMK, SCRIB,* and the developmental myosin heavy chains *MYH3* and *MYH8* in the Myoblast/Myocyte Signature,^37^^,^^38^^,^^39^^,^^40^^,^^41^ and *MYH2, INSR,* and *NOS1* in the Myonuclear Signature.^41^^,^^42^^,^^43^ However, other canonical markers such as *MYOD1* are not included due to expression in both the satellite cell and myoblast/myocyte groups. Similarly, several surface markers that have been used to isolate satellite cells in previous work such as CD20 and CAV1,^44^ were excluded based on transcript expression in other cell types such as smooth muscle and immune cells. These signatures also contain genes with little or no previous characterization in muscle. For example in the Satellite Cell Signature is *NPPC* that encodes a preproprotein that is proteolytically processed to generate multiple protein products including the cardiac natriuretic peptides CNP-53, CNP-29 and CNP-22^45^ and *PDE10A* that encodes a cyclic nucleotide phosphodiesterase that regulates cyclic nucleotides for signal transduction.^46^ Of particular interest in the Myoblast/Myocyte Signature are *SYTL2* and *LRRN1*, both of which have been identified as putative MYOD target genes^47^ but not characterized further in myogenic cells to our knowledge. While the functional relevance of these genes requires further investigation, their specificity is of value in identification and marker studies.

These signatures were validated in three different contexts: in bulk RNA-sequencing data of rodent muscle regeneration capturing known fluctuations in cell populations, in two independent single-cell datasets from human muscle, and through paired transcriptomic/histological analysis. First, using muscle post-injury transcriptomic dataset from rat, we show that the expression of these signatures reflects changes in cell populations that occur during early muscle regeneration when active myogenesis is occurring. This demonstrates the ability of these signatures to inform on changes in their respective cell populations in a bulk tissue setting. As the signatures were generated from murine single-cell data, we next confirmed their relevance to human using two independent single cell datasets from human skeletal muscle. The Satellite Cell and Myonuclei Signatures were powerful discriminators of cell identity in both datasets. Finally, we establish that changes in the expression of genes in the Satellite Cell Signature were correlated with the number of PAX7-containing satellite cells using paired transcriptomic and histological analyses from the same human muscle biopsies.

Our signatures use differential gene expression to delineate populations from bulk transcriptomic data. An alternative strategy in the form of a deconvolution method has also been developed to report on cell-type proportions in bulk transcriptomic data from human muscle.^25^ However, myoblast and myocyte populations were not included in this algorithm. Furthermore, we found that the expression of many genes used as the reference profile for satellite cells in this algorithm are not unique to satellite cells in the De Micheli et al.^26^ human single-cell dataset and the McKellar et al.^24^ murine dataset (data not shown), possibly due to either smaller numbers of cells assayed and/or because the reference gene lists used to develop the algorithm were based on data from a single donor.

Elevated expression of the Satellite Cell and Myoblast/Myocyte Signatures in patients with DMD is consistent with previous reports of elevated satellite cell numbers in DMD muscle through histological quantification^48^^,^^49^ and active muscle regeneration,^50^^,^^51^^,^^52^ providing further confirmation that our signatures measure biologically relevant changes. Importantly, these gene signatures also provide tools to gain insight into satellite cell activity and myogenesis in any bulk muscle transcriptomic dataset, including conditions in which data on myogenesis/regeneration is more limited, such as DM1 and FSHD. An increased number of satellite cells in distal muscles (tibialis anterior) of patients with DM1 compared to proximal muscles and healthy controls has been reported.^53^ Together with active muscle regeneration in DM1,^54^ this is consistent with the elevated Myoblast/Myotube Signature expression in DM1 patient-derived RNA-sequencing data.

A previous study reported no difference in the number of PAX7-expressing satellite cells in muscle biopsies from ten patients with FSHD compared to healthy controls.^55^ These muscles were not examined by MRI prior to biopsy, and the majority were ranked low by pathology score. This is consistent with the lack of change in expression of the Satellite Cell Signature observed in TIRM^–^ vastus lateralis muscles at the group level. However, when the TIRM^–^ muscle samples are segregated based upon the overall clinical severity of the FSHD donor, increased expression of both the Satellite Cell and Myoblast/Myocyte Signatures was found in the more clinically severe TIRM^–^ muscle samples compared to both those classified with mild clinical severity, and controls. This suggests that some muscles that appear “unaffected” based on TIRM hyperintensity may still have subtle pathological changes. Satellite Cell and Myoblast/Myocyte Signature expression was further increased in TIRM^+^ muscle, which indicates increased myogenesis as FSHD pathology progresses and is consistent with our recent report that muscle regeneration in patients with FSHD correlates with advancing pathology.^27^

In summary, we present gene signatures specific to Satellite Cells, Myoblasts/Myocytes, and Myonuclei that are suitable to apply to bulk transcriptomic datasets. Expression of these signatures has been validated in bulk transcriptomes of early stages of rat muscle regeneration, in human single cell datasets, and through direct correlation of signature expression with the histological examination of satellite cells. The consistent pattern of increased expression of genes pertaining to mononuclear myogenic progenitor cells (Satellite Cells and Myoblast/Myocytes) across various forms of muscular dystrophy reflects active repairing/regenerating muscle tissue. These signatures also provide a tool to examine satellite cell activity across other forms of muscle disease and may contribute to the classification of satellite cell-opathies.^15^^,^^16^ Future work will determine whether our signatures have the resolution to detect subtler changes in satellite cell activity, for example following different exercise regimes. Thus, these gene signatures provide a relatively simple means to inform on myogenic cell populations from bulk muscle sequencing data, allowing further investigation of satellite cells activity and muscle regeneration.

### Limitations of study

Our gene signatures were derived from murine single cell transcriptomic data, and then the Satellite Cell Signature was optimized using two independent single cell transcriptomic datasets from human muscle. However, the human datasets did not contain sufficient numbers of cells actively undergoing myogenesis to allow similar validation of the Myoblast/Myocyte signature. Thus, the same gene selection thresholds used to establish the Satellite Cell Signature were also used to generate the Myoblast/Myocyte signature. While many genes with known roles in myogenesis were identified in this way, as the number of human single cell datasets increases it may be possible to further optimize the Myoblast/Myocyte signature.

The relatively small size of the Satellite Cell Signature (10 genes) maximizes its cell-type specificity within skeletal muscle. A limitation is that it may not be well-represented in certain datasets, restricting its use, as was found with the Wang et al. DM1 dataset^34^ where only 3/10 genes were present (Figure 7B).

Finally, the aim of generating these gene signatures is to gain information on satellite cell activity from bulk transcriptomic data. However, discrepancies between RNA expression and protein levels for some genes can occur. Thus, examination of muscle biopsies by histological/immunolabelling (for example) remains valuable to validate satellite cell activity, as well as to provide additional information such as the distribution of areas undergoing active regeneration and overall tissue morphology.

## STAR★Methods

### Key resources table

**Table:** 

REAGENT or RESOURCE	SOURCE	IDENTIFIER
**Antibodies**		

Mouse monoclonal anti-PAX7 primary antibody	DSHB	PAX7-s; AB_528428
Goat anti-mouse Alexa Fluor 594 secondary antibody	ThermoFisher	A11005; AB_2534073

**Biological samples**		

Human skeletal muscle tissue sections	This study	N/A

**Chemicals, peptides, and recombinant proteins**		

Hoechst 33342	ThermoFisher	H3570
Wheat germ agglutinin, AlexaFluor 647-conjugated	Invitrogen	W32466

**Deposited data**		

Human reference genome build 38, GRch38	Ensembl	https://ftp.ensembl.org/pub/release-111/fasta/homo_sapiens/cdna/
Banerji et al.^28^ RNA-sequencing data	European genome-phenome archive	EGAS00001007350
Wong et al.^29^ RNA-sequencing data	Fred Hutch Cancer Center Github repository	https://github.com/FredHutch/RWellstone_FSHD_muscle_biopsy/
Ren et al.^31^ RNA-sequencing data	Gene Expression Omnibus	GSE171243
Rubenstein et al.^25^ single cell RNA-sequencing data	Gene Expression Omnibus	GSE130646
De Micheli et al.^26^ single cell RNA-sequencing data	Gene Expression Omnibus	GSE143704
Wang et al.^34^ RNA-sequencing data	Wang et al.^34^	Table S5
Khairallah et al.^33^ RNA-sequencing data	Khairallah et al.^33^	Table S2

**Software and algorithms**		

TrimGalore! [v0.6.6]	Martin et al.^56^	RRID:SCR_0118417
cutadapt [v1.15]	Martin et al.^56^	RRID:SCR_0118471
FastQC [v0.11.5]	Andrews et al.^57^	RRID:SCR_014583
MultiQC [v1.11.dev0]	Ewels et al.^58^	RRID:SCR_014982
Salmon [v1.4.0]	Patro et al.^59^	RRID:SCR_017036
tximeta [v1.16.1]	Love et al.^60^	https://bioconductor.org/packages/release/bioc/html/tximeta.html
DESeq2 [v1.38.3]	Love et al.^61^	RRID:SCR_015687
pROC [v1.18.0]	Robin et al.^62^	RRID:SCR_024286

### Resource availability

#### Lead contact

Further information and requests for resources and reagents should be directed to the lead contact, Peter S. Zammit (peter.zammit@kcl.ac.uk).

#### Materials availability

This study did not generate new unique reagents.

#### Data and code availability

• This paper analyzes existing, publicly available data. These accession numbers for the datasets are listed in the key resources table.
• All original code is available from the authors upon request.
• Any additional information required to reanalyse the data reported in this paper is available from the lead contact upon request.

### Experimental model and study participant details

#### Human muscle sections

Human muscle sections were obtained from the cohort of 24 FSHD patients and 11 non-FSHD control individuals described previously,^28^ with the exception of 3/11 controls from which histological sections were unavailable. An additional 2/24 TIRM+ samples could not be analyzed due to an insufficient number of myofibers. All patients were investigated between 2019 and 2021 at the Radboud University Medical Center (Nijmegen, The Netherlands) Neurology Outpatient clinic. Subjects provided written informed consent and the study received regional medical ethical committee approval (CMO Arnhem-Nijmegen).

### Method details

#### Generation of gene signatures

The McKellar et al.^24^ integrated dataset of 365,011 murine cells, as well as the dataset containing the subset of 84,383 myogenic cells, were downloaded from http://scmuscle.bme.cornell.edu and analyzed in R (v4.2.2) using Seurat^63^ (v4.3.0). Cluster annotations for non-myogenic cells and myonuclei were inherited from the authors’ original assignments. For myogenic precursors, cells were grouped by bins generated through the authors’ pseudotime analysis of the myogenic compartment. Bins 4–5 were termed “Satellite Cells”, and bins 6–18 were renamed “Myoblasts/Myocytes”.

When filtering differentially expressed genes from the McKellar et al.^24^ dataset for the Satellite Cell signature, several thresholds were evaluated for each of three gene selection parameters as follows: 1.5-fold, 1.75-fold, or 2-fold-change increase in gene expression in satellite cells, expression detected in a maximum of 5%, 10%, or 15% of all other cells, and expression detected in a maximum of 20%, 30%, or 40% of cells within any other individual cluster. Of the resulting 27 gene lists generated from each combination of these selection parameters, only those containing a minimum of 10 genes were further considered to prevent over-reliance on a small number of features and ensure the signature was robust to differences in coverage and detection across technologies.

We then determined the gene list that best discriminated satellite cells from non-satellite cells in the independent De Micheli et al.^24^ (GSE143704) human dataset using Receiver operating characteristic (ROC) curve analysis. This list was defined as the Satellite Cell signature (10 genes). The same selection criteria (>1.5-fold increase in expression in the given cell type, expression in <10% of all other cells and <30% of cells within any other individual cluster) were then used to identify genes expressed predominantly by myoblasts/myocytes (25 genes) and type I/IIa/IIx myonuclei (68 genes) in the McKellar et al.^24^ dataset. Two genes from the Myonuclear signature were excluded due to lack of human ortholog, leaving 66 genes in the signature (Table 1, Table S1). For UMAP plots colored by signature expression, expression was calculated by summing the log-transformed counts of genes in a signature within each cell.

#### Analysis of bulk RNA-sequencing datasets

Raw counts from rodent muscle regeneration from Ren et al.^31^ were downloaded (GSE171243) and log-normalized using the DESeq2 package (v1.38.3).^61^ Expression scores were calculated as the sum of the log-normalised gene level counts of a signature and presented as difference from the T0 (non-injured) average. Significance was assessed by one-way ANOVA with Tukey’s post-hoc test.

For the Banerji et al.^28^ human dataset (European Genome-phenome archive EGAS00001007350), raw reads were trimmed using TrimGalore! (v0.6.6) and cutadapt (v1.15) to remove Illumina Sequencing Adapters from the 3′ end, and FastQC (v0.11.5) and MultiQC (v1.11.dev0) to confirm quality of sequence data. Raw reads were mapped to the human transcriptome using Salmon^59^ (v1.4.0) with human genome assembly GRCh38 and v104 annotations from Ensembl and summarised to the gene level in R using tximeta^60^ (v1.16.1). The Wong et al.^29^ human dataset was downloaded as a DESeq2 object from (https://github.com/FredHutch/RWellstone_FSHD_muscle_biopsy/). For both datasets, log-normalised gene level counts were calculated using DESeq2, and signature expression was calculated as the difference between the sum of log-transformed counts of genes in the signature within each sample and the control average. Significance was assessed in R by two-tailed Wilcoxon ranked-sum test (paired for TIRM^–^ versus TIRM^+^, unpaired for other comparisons) for Banerji et al.^28^ and by one-way ANOVA with Tukey’s post-hoc test or Kruskal-Wallis test with Dunn’s post-hoc test for Wong et al.^29^

For the DM1 and DMD human datasets, normalized count matrices were downloaded from the original publications (DMD: Table S2 from Khairallah et al.^33^ and DM1: Table S5 from Wang et al.^34^). Signature expression was calculated as the difference between the sum of log-normalised gene level counts of all genes in a signature within each sample following subtraction of the control average. Significance was assessed in R by two-tailed Wilcoxon ranked-sum test.

All datasets contained at least 75% of genes in each of the three signatures except for the Wang DM1^34^ dataset, which only contained 3/10 genes in the Satellite Cell signature. When a gene was absent from a dataset it was excluded from the signature for that particular analysis.

#### Analysis of single cell RNA-sequencing datasets

Raw data from Rubenstein et al.^25^ human single cell data was downloaded from the Gene Expression Omnibus (GSE130646) in the format provided by the authors and merged in Seurat.^63^ Raw counts and metadata from De Micheli et al.^26^ were downloaded from the Gene Expression Omnibus (GSE143704). Seurat^63^ was used to process data, using the authors’ original analysis parameters. For both datasets, Seurat^63^ was used to merge samples and normalize counts and authors’ original cell annotations were maintained for ROC analysis. ROC curve analysis and AUC calculations were performed using the pROC package (v1.18.0)^62^ to evaluate signature expression as a binary classifier of cell identity (per authors’ cell type annotations), where signature expression is the sum of the log-transformed counts of all genes in the signature within each cell.

#### Immunolabeling

The MRI-guided muscle biopsies used here were collected previously, as described in Banerji et al. 2023.^28^ Muscle biopsies were immediately snap frozen in liquid nitrogen cooled isopentane and stored at −80°C. Serial muscle cryosections of 6 μm were obtained from each sample using a Leica CM3050S cryostat at −23°C and stored at −20°C.

For staining and immunolabelling, sections were washed 3x in Hanks’ balanced salt solution (HBSS) and stained with Alexa Fluor 647 conjugated wheat germ agglutinin (WGA) (ThermoFisher) diluted 1/50 in HBSS for 10 min at room temperature (RT). Sections were then permeabilized in 0.5% Triton/PBS for 10 min at room temp and then blocked in 5% goat serum/2% BSA/0.1% Triton/PBS for 1 h. Sections were incubated in anti-PAX7 (DSHB) diluted 1/5 in blocking solution at 4° overnight, washed and then incubated in goat anti-mouse Alexa Fluor 594 at 1/400 in blocking solution at RT for 1 h. Nuclei were stained for 10 min at RT in 1:1000 Hoechst33342 (ThermoFisher) and mounted in mounting medium (ibidi). Entire sections were imaged on a Zeiss AxioScan Z1 Automated Slide Scanner at 20× magnification. The number of PAX7-expressing nuclei per total fiber number was quantified using ImageJ, and Spearman’s correlations between expression of the Satellite Cell signature in paired RNA-sequencing samples were computed in R. The PAX7 monoclonal antibody developed by A. Kawakami was obtained from the Developmental Studies Hybridoma Bank, created by the NICHD of the NIH and maintained at The University of Iowa, Department of Biology, Iowa City, IA 52242.

### Quantification and statistical analysis

All statistical methods used in this study are listed in the figure legends, as well as in the relevant subheadings of the previous “method details” section.

## Supplemental information

Table S1. Description of genes in the Satellite Cell, Myoblast/Myocyte, and Myonuclear Signatures, related to Table 1
